# Bitters: Time for a New Paradigm

**DOI:** 10.1155/2015/670504

**Published:** 2015-05-14

**Authors:** Michael K. McMullen, Julie M. Whitehouse, Anthony Towell

**Affiliations:** ^1^Life Force Research, 45930 Ljungskile, Sweden; ^2^Department of Life Sciences, Faculty of Science and Technology, University of Westminster, London W1B 2HW, UK; ^3^Department of Psychology, Faculty of Science and Technology, University of Westminster, London W1B 2HW, UK

## Abstract

In plant-based medical systems, bitter tasting plants play a key role in managing dyspepsia. Yet when it comes to defining their mechanism of activity, herbalists and pharmacologists are split between two theories: one involves cephalic elicited vagal responses while the other comprises purely local responses. Recent studies indicate that bitters elicit a range of cephalic responses which alter postprandial gastric phase haemodynamics. Caffeine and regular coffee (*Coffea arabica semen*, L.) increase heart rate whereas gentian (*Gentiana lutea radix*, L.) and wormwood (*Artemisia absinthium herba* L.) increase tonus in the vascular resistance vessels. Following meals increased cardiac activity acts to support postprandial hyperaemia and maintain systemic blood pressure. The increased vascular tonus acts in parallel with the increased cardiac activity and in normal adults this additional pressor effect results in a reduced cardiac workload. The vascular response is a sympathetic reflex, evident after 5 minutes and dose dependent. Thus gentian and wormwood elicit cephalic responses which facilitate rather than stimulate digestive activity when postprandial hyperaemia is inadequate. Encapsulated caffeine elicits cardiovascular responses indicating that gastrointestinal bitter receptors are functionally active in humans. However, neither encapsulated gentian nor wormwood elicited cardiovascular responses during the gastric phase. These findings provide the platform for a new evidence-based paradigm.

## 1. Introduction

Bitter tasting herbs, commonly referred to as bitters, are used in many cultures [1–4] to support upper digestive activity; yet there is no consensus regarding a mechanism by which the chemosensory stimulation of taste receptors could enhance digestion. Results from our research group indicate that a novel mechanism is involved. Namely, some bitter tastants elicit a cephalic response increasing peripheral vascular resistance (PVR) [5]. During digestion, postprandial hyperaemia (PPH) places demands of the cardiovascular system (CVS) which are met by increased cardiac activity so as to prevent postprandial hypotension [6–10]. The increased PVR supports this cardiac activity and facilitates adequate PPH.

## 2. Theories of Bitter Activity

In the literature there are four models, two common and two minor, proposed to explain the effect of bitters on digestion.

- CEPHALIC VAGAL REFLEX: stimulation of the oropharyngeal bitter receptors acts reflexively to increase saliva and vagal stimulation to the digestive organs.
- LOCAL REFLEX: stimulation of both oropharyngeal and gastrointestinal bitter receptors acts locally to increase digestive secretions.
- ALCOHOL: alcohol, rather than the bitter taste, improves digestion.
- HYPERAEMIA: the major influence of bitters on digestion is improved blood circulation in the abdominal organs.

### 2.1. Cephalic Vagal Reflex

This longstanding model was reiterated in a 19th century pharmacology text: “the appetite is sharpened because the gustatory nerves are stimulated; this reflexively leads to dilation of the gastric vessels and to an increase in the gastric and salivary secretions” [11]. The later work of Pavlov, on the autonomic nerve system, was considered to support this model [12].

A more recent description of this model is as follows: “bitter stimuli pass primarily by way of the glossopharyngeal nerve to a special group of cells in the cerebral cortex. The taste is interpreted there as bitter, and causes stimuli to be forwarded through the vagus nerve to both the salivary gland and the stomach…. This stimulation of the digestive processes enhances the appetite” [13]. This model is supported by German E Commission [14] and several European Medical Agency Assessment Reports:

1. gentian (*Gentiana lutea* L.): “the long known action of bitters, which increase the secretion of gastric juice and bile due to the stimulation of gustatory nerves in the mouth” [15];
2. wormwood (*Artemisia absinthium* L.): “it is long known that the bitter constituents stimulate the gustatory nerves in the mouth and increase the secretion of gastric juice and bile, thereby promoting appetite and digestion” [16].

However, not all German pharmacologists agree [17]. In fact, generally pharmacologists are split on this issue with many agreeing [2, 18–25] and many disagreeing [17, 26–29]. The latter maintain that this model is unproven [29]. This difference of opinion results from the simple fact that the bitters have not been scientifically investigated [26]. A significant reason for this absence of research is the lack of investigative tools [30] to assess the levels of digestive secretions and more particularly the content of the secretions. Additionally, even if cephalic stimulation by bitters increased the production of digestive enzymes, it is the actual presence of food that stimulates enzyme secretion [31]. Thus studies would need to include both bitters and foods, a significant procedural complication.

Some pharmacologists claim that “bitter principles act reflexively on the cardiovascular system causing a decrease of heart rate and cardiac stroke volume” as evidence to support the model of bitters increasing vagal activity [32]. However these claims, based on an earlier study [33], have been shown to be fallacious, as the effect on heart rate and stroke volume for bitters added to water is no different than the effect of plain water on these parameters [34]. In contrast to the notion that digestion would benefit from enhanced parasympathetic activity [32], digestion is widely considered to be a sympathetic activity [35].

It has also been argued that bitters only affect those with impaired digestion [25, 32] yet no mechanism is suggested by which this selection process could occur. Also the question remains: if bitters increase digestive secretions, why do consumers of bitter aperitifs do not suffer side effects resulting from excess secretion of stomach acid, pancreatic juices, and gall?

### 2.2. Local Stimulation

This simple model asserts that the stimulation of bitter receptors acts locally to increase digestive secretions. This is certainly true for the oropharyngeal cavity as it is well known that bitter and sour tastants increase saliva production. Regarding the gastrointestinal tract, the discovery of bitter receptors in the gastrointestinal tract tissue [36] suggests that bitters may elicit chemosensory responses throughout the entire digestive system.

The presence of functionally active gastrointestinal bitter receptors in humans has been established when it was demonstrated that encapsulated caffeine increased both arterial compliance and diastolic pressure [37]. Yet it remains to be demonstrated that sufficient receptor stimulation occurs in physiological situations, such as eating, when contact between the agonist and the receptor may be hindered by the presence of food. Additionally there is the problem of partial agonists and antagonists [38] whether using natural or isolated substances.

### 2.3. Alcohol

The American pharmacologist Tyler suggested that for aperitifs it was the alcohol rather than the tastants which were responsible for any improvements in digestion [13]. While various alcoholic beverages do delay gastric emptying [39–41], including aperitifs [42, 43], the amount of alcohol found in a single dose of bitters made from either fluid extracts or tinctures is minimal (0.5–1.5 mL) and unlikely to affect gastric emptying.

### 2.4. Hyperaemia

Both Hale White (1892) and Weiss (1988) have proposed that the bitters enhance blood circulation in the gut and thereby improve digestion. Hale White wrote that following the ingestion of bitters: “the appetite is sharpened because the gustatory nerves are stimulated; this reflexively leads to dilation of the gastric vessels” [11]. Similarly Weiss wrote “the appetite-inducing action of bitters is probably due to improved circulation in the abdominal organs” [18]. Furthermore, Weiss suggested that bitters increase sympathetic excitability over time and it was this repeated activation that produced a general tonic action.

This model is of interest because following studies in the 1980s and 1990s it was realised that the increased splanchnic circulation after eating, referred to as postprandial hyperaemia (PPH), is the limiting factor of the digestive process [6, 7, 9, 10]. PPH fuels the metabolic activity during digestion supplying nutrients for the following:

- the production of digestive secretions;
- the movement of chyme;
- the absorption of nutrients;
- the movement of nutrients away from the intestinal wall;
- the removal of metabolic wastes.

PPH extends the systemic circulation, potentially reducing blood pressure (BP) and peripheral vascular resistance (PVR). To avoid hypotension cardiac activity increases, both heart rate [9] (HR) and cardiac contraction force (*dP*/*dt*) [5] increase, raising cardiac output (CO) and preventing a drop of BP and PVR [9]. An inadequate postprandial cardiac response leads to both digestive problems and postprandial hypotension [44]. Postprandial hypotension is a major problem amongst the elderly [45] and a predictor of all-cause mortality in the low-care elderly [46].

## 3. Experimental Findings

In studies using the bitters roasted coffee (*Coffea arabica semen*, L.) drinks, both regular (circa 130 mg caffeine) and decaffeinated, 133 mg encapsulated caffeine (BP) [37, 47], gentian (*Gentiana lutea radix*, L.) and wormwood (*Artemisia absinthium herba*, L.) [5], the impact of bitters was assessed on gastric phase haemodynamics.

**Figure 1:**
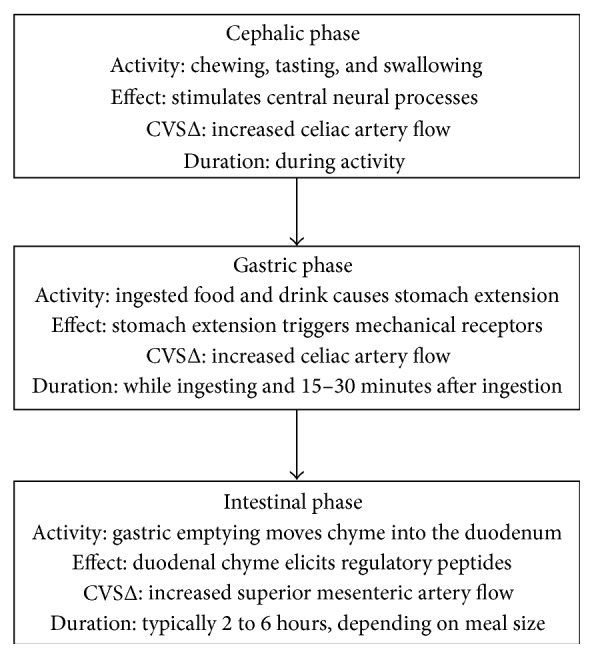
The three phases of digestion. CVSΔ: cardiovascular system change.

There are three phases of digestion [31, 48] (Figure 1).

1. Cephalic phase where chewing, tasting, and swallowing stimulate neural processes to increase blood flow in the celiac artery.
2. Gastric phase where the presence of either food or fluids produces stomach extension. Stomach extension triggers mechanical receptors which elicits increases of the celiac blood flow. This period begins when food enters the stomach and extends to when gastric emptying starts, usually 15–30 minutes after ingestion has ceased. It is during this period when postprandial hypotension most commonly occurs [8].
3. Intestinal phase begins with gastric emptying and continues until the meal is fully digested. During this phase the blood flow in the superior mesenteric artery increases in a manner governed by the food composition. Maximum flow increase is greatest for carbohydrates and least for proteins with fats in the middle [49] although the total response may not vary between types of meals [50]. Neither water (room temperature or cold) nor sham feeding affects the mesenteric flow [49].

### 3.1. Coffee and Caffeine

Compared to control, encapsulated caffeine increased arterial compliance and diastolic BP in the 10–15 postingestion period, that is, just after the capsules opened [37]. As noted above, this finding indicates the presence of functionally active gastrointestinal bitter receptors in humans. In the later period 25–30 minutes, CVS changes were absent. The difference between the impacted CVS parameters in the two periods is likely due to the fact that caffeine, being rapidly and completely absorbed from the gut [51], is likely absent from the gut during the 25–30 minutes period. Notably there is no residual effect on the vasculature.

**Figure 2:**
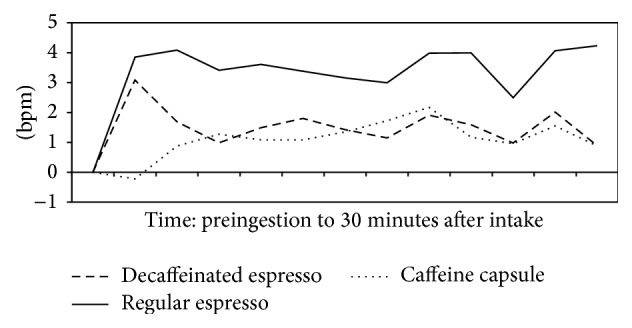
Postingestion changes in heart rate minus placebo postingestion changes. bpm: beats per minute.

Compared to the room temperature control, hot preparations of regular coffee and decaffeinated espressos (67 mL) increased HR immediately after ingestion [37]. This is expected as hot water is known to increase HR [52]. Additionally, for the regular coffee, HR increases continued to the 30-minute mark (Figure 2). A follow-up study [47] reported that decaffeinated coffee with addition of caffeine produces similar results to caffeine.

The increase in HR, without changes in dP/dt, PVR, or baroreceptor sensitivity, suggests that the caffeine is eliciting vagal withdrawal, that is, reducing the vagal brake on the autonomic nerve system, for at least 30 minutes after the ingestion of regular coffee.

These findings indicate that the bitter compound caffeine is likely the active substance in regular coffee eliciting HR increases. In contrast the quinides, responsible for coffee's bitter taste, elicited no CVS responses. This variation indicates that dissimilar bitters may elicit different responses and so the hedonistic quality of bitterness is not an indicator of particular cephalic chemosensory responses.

The contrasting findings, that the chemosensory stimulation of oropharyngeal receptors by caffeine elicits increased HR while the chemosensory stimulation of gastrointestinal receptors by caffeine elicits increased AC, can be explained by the varying neural connections of the oropharyngeal and the gastrointestinal receptors. The oropharyngeal receptors have neural connections terminating in the rostral area of the nucleus tractus solitarius, whereas the gastrointestinal receptors have neural connections terminating in the caudal area of the nucleus tractus solitarius. Alternatively as caffeine is an agonist for 5 bitter receptors (*h*TAS2R: 7, 10, 14, 43, and 46) [53] it may be stimulating different receptors or constellations of receptors in the different tissues. Either way, the differing CVS responses indicate it cannot be assumed that a response elicited by a bitter agonist in one tissue can predict the response to the same agonist in another tissue.

### 3.2. Gentian and Wormwood

Gentian and wormwood are some of the most commonly used bitters in Europe [18, 29] and monographs of both drugs have recently been prepared by the European Medical Agency [15, 16, 54, 55]. The impact of these drugs on gastric phase haemodynamics was investigated in two parts.

In the first part, 100 mL room temperature water and capsules containing 1000 mg plant material were compared with placebo control during the 10–15-minute postingestion period, that is, just after the capsules opened. In contrast to the caffeine capsules, neither gentian nor wormwood altered CVS parameters.

In the second part, 1 mL of fluid extracts (40% alcohol) prepared from 500 mg and 1500 mg of both drugs was administered in 100 mL room temperature water. Compared to the control, both drugs elicited CVS changes in the 5–10-minute postingestion period. However, to fully appreciate the impact of the drugs it is necessary to recognise the impact of the control. The control elicited increases in dP/dt resulting in increased SV, systolic BP, and diastolic BP (see Table 1). The reduced HR is likely also the result of the increased dP/dt. The increased BP will decrease HR via the baroreflex activity [56]. With gentian and wormwood fluid preparations, the larger doses of both drugs and to lesser degree the smaller dose of gentian increased PVR or vascular tonus. This led to a reduction of cardiac activity (decreased SV and CO) without altering BP, presumably via baroreflex activity. Hence by increasing the vascular tone the bitters are complimenting the cardiac activity, which is compensating for the PPH. The net effect of the increased vascular tone is a reduction in cardiac workload. As these changes were observed for the fluids but not the capsules, it can be concluded that the gentian and wormwood are increasing PVR by eliciting cephalic chemosensory reflex responses.

## 4. Methodological Considerations

**Figure 3:**
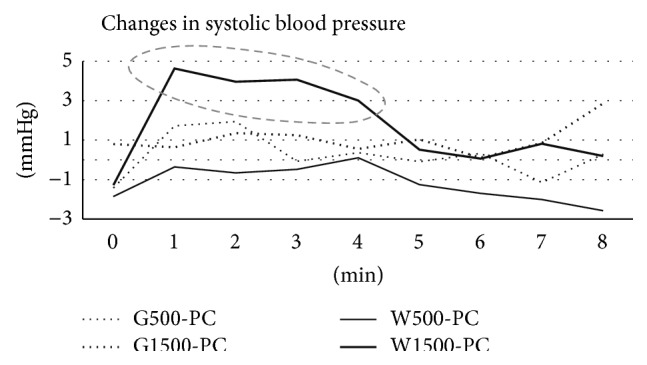
Postingestion placebo-controlled changes from baseline for systolic blood pressure following the intake of four bitter preparations. The sharp increase in blood pressure (broken ellipse) for W1500 during the first minute and extending to 5 minutes is likely the result of a startle or alerting response to the tastant. G500: extract produced from 500 mg gentian, G1500: extract produced from 1500 mg gentian, W500: extract produced from 500 mg wormwood, W1500: extract produced from 1500 mg wormwood, PC: placebo control.

Whether designing or assessing studies on the impact of tastants on CVS, there are physiological, perceptual, statistical, and parameter recording issues which need to be considered. Firstly the impact of swallowing dominates the initial minutes of postingestion period. Secondly, a high intensity tastant may produce a startle [57] or alerting [58] reaction which may last up to 5 minutes (Figure 3). Instances in the literature where startle reactions may have occurred include the effects of quinine sulphate on the CVS [59] and gastric emptying [60, 61]. Thirdly, the necessity of a control condition to assess interventions in nonpathological populations is critical [62], and in particular comparisons to baseline are inappropriate [63]. Due to the inherent difficulty in blinding the presentation of tastants, between-participant designs are often preferable to within-participant designs. Fourthly, combining measures from both the gastric- and intestinal phases produces uninterpretable results because the two phases are physiologically distinct. Lastly applying repeated measures ANOVA to a series of nonindependent measures is incorrect. Repeated measures ANOVA is only valid when applied to measures obtained from independent readings, such as the same test group tested with multiple treatments on separate occasions. Thus it is inappropriate to use this analysis technique for serial measurements such as readings at 30, 60, and 90 minutes from the same test session, unless independence can be demonstrated. For serial measurements the statistically legitimate procedure is to produce summary measures from the readings prior to analysis [64, 65]. This is a surprising common mistake and a review of 125 publications reported that post hoc *t*-tests and repeated measure ANOVAs were incorrectly applied in 56% and 52% of cases, respectively [66]. CVS studies failing to report dP/dt measures can be considered incomplete while short-term changes of heart rate variability scores are misleading unless dP/dt can be shown to be fixed [67].

## 5. Discussion

The studies outlined above provide a platform on which we can build a new evidence-based model, on the impact of bitters on humans, grounded on a physiological mechanism.

Some bitter tastants elicit cephalic reflex autonomic and CVS responses whereas others appear not to, such as the quinides in coffee. Consequently there is no universal bitter effect [25], as different bitters elicit a range of cephalic responses, or even none at all.

Some bitter tastants elicit cephalic responses increasing HR, the mechanism of which is likely due to vagal withdrawal for example, caffeine. This is consistent with caffeine's reputation as a human activator, although the chemosensory pathway is novel. It is even possible that at the dose of circa 130 mg this response occurs without hedonistic recognition [68]. Despite altering postprandial haemodynamics, it remains to be determined whether the increased HR affects PPH. Currently the mechanism by which regular coffee [69], but decaffeinated coffee [70], increases gastric emptying is unknown but may involve cephalic responses.

Some bitter tastants elicit cephalic responses increasing PVR, namely, gentian and wormwood. This change in postprandial haemodynamics is likely to impact inadequate PPH by increasing BP in situations where the cardiac output is insufficient. Thus this type of bitter assists digestion, rather than stimulating digestion as suggested by many authors [17, 32]. In fact, these bitters can be described as eupeptics (from Greek* eu*: well and* pepis*: digestion) promoting gastric juice secretion and facilitating digestion [27]. By implication, this finding suggests that bitters which elicit increased PVR may be of use in cardiac disorders, particularly those involving digestion such as postprandial angina [44, 46]. This finding also helps us to understand why these bitters help those with poor digestion but do not affect those with normal digestion [25, 29, 32].

A minimum dose of bitters is essential to elicit cephalic responses, for example, wormwood, and the magnitude of a response is dose dependent for example, gentian. Additionally, the hedonist perception of bitterness is not an indicator of a cephalic response. This finding contradicts the view that bitters only need to be tasted to be therapeutically active [25]. Furthermore the finding suggests that drop doses, such as 5–10 drops of 1 : 5 tinctures, recommended by some authors [25], are likely to be ineffective. Rather these findings provide evidence for the single doses, prepared from 1000 mg gentian or 1000 mg wormwood, recommended by the German E Commission [14] and the European Medical Agency monographs [54, 55].

The onset of increased HR and PVR is circa 5 minutes. This contrasts with the view that cephalic responses are slow [25] and therefore bitters should be taken 15–30 minutes prior to eating [2, 25]. Thus there appears to be little reason not to take bitters directly before, during, or even after a meal. It has been proposed that the cephalic response is limited to 30 minutes [27]; this remains to be established and ideally would require a study incorporating a meal. In the above studies with gentian and wormwood, several participants spontaneously reported that the bitter taste remained for in excess of 30 minutes. Other authors note that a bitter aftertaste may be present hours and even days after exposure [71]. Two publications report bitter combinations improving gastroparesis (inadequate gastric emptying) in pathological groups [72, 73]. Despite the limitations of these studies, the results suggest that the effect of these combinations is continuing well into the intestinal phase of digestion. Whether these combinations are increasing PPH or working directly on the gastric emptying process is unclear, but it is plausible that improving reduced PPH would encourage normal gastric emptying.

The fast onset suggests that in acute, and even some chronic, conditions dosage could be repeated at 30-minute intervals or even more often when required. Although the European Medical Agency monograph limits the use of wormwood [55], due to the thujone content, the accompanying Assessment Report notes an absence of thujone in percolated fluid extracts (30% alcohol) [16]. This report has also been criticised as basing recommendations on outdated animal studies and producing overly conservative conclusion regarding thujone [74]. Modern toxicity research indicates that it would take between 2 and 20 cups of wormwood tea to reach the maximum acceptable daily intake level [74] assuming that wormwood tea extracts 100% of the thujone in the dried herb [16]. There are no similar limitations for gentian [54].

The finding that encapsulated caffeine elicited gastric CVS responses indicates that tastants in the gastrointestinal tract are capable of rapidly eliciting systemic responses. The mechanism for caffeine responses is likely via vagal connections with neural relays in the CVS section of the nucleus tractus solitarius. This possible mechanism is supported by recent work indicating that more neural relays exist in taste transduction than previously realised [75]. The fact that secondary plant metabolites in the gastrointestinal are capable of eliciting autonomic and CVS responses indicates how exposed our homeostatic and circulatory systems are to dietary compounds as well as other substances formed in the gut either during digestion of foods or by the microbiota.

## 6. Conclusions

It is now possible to categorise bitters into functional subgroups rather than chemical subgroups based on mechanism of action. The eupeptics, gentian, and wormwood elicit cephalic responses via the sympathetic system, increasing peripheral vascular resistance. There is no evidence that either of these eupeptics enhance vagal transmissions, even if heart rate decreases. The increased cardiac activity in response to postprandial hyperaemia is complimented by the enhanced vascular tonus. This reduces the load on the heart and so the eupeptics may have therapeutic uses outside of their digestive usage, particularly regarding postprandial cardiac insufficiency. Although encapsulated gentian and wormwood did not elicit responses during the gastric phase of digestion, they may elicit responses during the intestines-phase of digestion. Gentian and wormwood may be used, both to prevent dyspepsia and to relieve dyspepsia, in fluid doses containing circa 1000 mg of the dried drug. In chronic conditions, during which the digestive organs have experienced a period of insufficient blood flow or ischaemia, both local and systemic tonic effects can be expected to result from the improved splanchnic blood flow. These eupeptics have a fast onset and dosage can be repeated as required. The low toxicity favours a user-regulated regime of administration.

## Figures and Tables

**Table 1:** Cardiovascular changes in the gastric phase of digestion.

Condition	HR	*dP*/*dt*	SV	CO	PVR	AC	S.BP	D.BP
Control	↓^∧^	↑^∧∧^	↑^#^	0	0	0	↑^∧∧^	↑^∧∧^
G500	0	0	↓^∗^	↓^∗^	↑^†^	0	0	0
G1500	0	0	↓^∗^	↓^∗∗^	↑^∗∗^	0	0	0
W500	0	0	↓^∗∗^	↓^∗∗^	↑^∗∗^	0	0	0
W1500	0	0	0	0	0	0	0	0

HR: heart rate, *dP*/*dt*: contraction force, SV: stroke volume, CO: cardiac output, PVR: peripheral vascular resistance, AC: arterial compliance, S.BP: systolic blood pressure, D.DP: diastolic blood pressure, G500: gentian 500 mg, G1500: gentian 1500 mg, W500: wormwood 500 mg, W1500: wormwood 1500 mg. ↑: increase, and ↓: decrease; ^#^0.05 ≤ *p* < 0.010, ^∧^ *p* < 0.05, ^∧∧^ *p* < 0.001 pre-post ingestion comparisons for the control condition; ^†^0.05 ≤ *p* < 0.010, ^∗^ *p* < 0.05, ^∗∗^ *p* < 0.001 placebo-controlled pre-post ingestion comparisons.
